# College Members whose deaths were reported at Council meetings between October 2015 and October 2016

**DOI:** 10.1192/pb.41.1.60

**Published:** 2017-02

**Adabie**, Kenneth Horn, *Affiliate*, Croydon, London, UK

**Al-Hindawi**, Amir Abdul Amir Ali, *Affiliate*, Rotherham, South Yorkshire, UK

**Arshad**, Muhammad, *Affiliate*, Cheadle, Cheshire, UK

**Baker**, Ronald Stewart, *Fellow*, Southwold, UK

**Barker**, Montagu Gordon, *Fellow*, Bristol, UK

**Barker**, Philip, *Fellow*, Calgary, Alberta, Canada

**Betts**, Timothy, *Fellow*, Birmingham, UK

**Bhate**, Suryakant Ramji, *Fellow*, Newcastle upon Tyne, UK

**Browne**, Elizabeth Foster, *Foundation Member*, London, UK

**Burrows**, Graham Dene, *Fellow*, Richmond, Victoria, Australia

**Burvill**, Peter Walter, *Fellow*, Como, Western Australia, Australia

**Carney**, Michael William Patrick, *Fellow*, Bathampton, Bath, UK

**Cheyne**, Alexander Ian, *Fellow*, Kippen, Stirling, UK

**Choudhary**, Prasoon Chandra, *Fellow*, Aberdare, UK

**Davies**, Stephen Lewis, *Member*, Breaston, Derby, UK

**Denson**, Raymond, *Member*, Thunder Bay, Ontario, Canada

**Discombe**, Anne-Marie, *Member*, Glasgow, UK

**Edmonstone**, Yvonnne Grace, *Fellow*, Inverness, UK

**El Gorashi**, Gamal El Din Suleiman, *Member*, Canterbury, UK

**Fielder**, Michael Hugh, *Member*, London, UK

**Forrest**, Derek William, *Fellow*, Wirral, UK

**Frame**, Archibald Hamilton, *Fellow*, Glasgow, UK

**Galea**, Abraham, *Fellow*, Mosta, Malta

**Galley**, Margaret Walton, *Foundation Affiliate*, Castletown, Isle of Man

**Galley**, Wilfred, *Foundation Affiliate*, Castletown, Isle of Man

**Gay**, Martyn John, *Fellow*, Newton Poppleford, Sidmouth, UK

**Gosall**, Gurpal Singh, *Member*, Brockhall Village, Old Langho, Blackburn, UK

**Graham**, Alexander John, *Fellow*, Law, Carluke, UK

**Gundy**, Greville Henry, *Member*, South Croydon, London, UK

**Hall**, Peter, *Fellow*, Upton upon Severn, UK

**Halpenny**, John Vincent, *Member*, Swords, Co Dublin, Ireland

**Hanmanthraya**, Bheemraya, *Member*, Durham, UK

**Hassan**, Imad Mohamed Babiker, *Affiliate*, London, UK

**Hervey**, Morag Stuart, *Fellow*, Wells, UK

**Hinton**, John Mark, *Fellow*, Leigh, Sherborne, UK

**Hocking**, Frederick H., *Fellow*, Melbourne, Victoria, Australia

**Hordern**, Anthony, *Fellow*, North Turramurra, New South Wales, Australia

**Hughes-Roberts**, Huw Elfyn, *Fellow*, Prestatyn, UK

**Jones**, Eve, *Member*, Mellor, Blackburn, UK

**Kay**, David William Kilbourne, *Fellow*, Newcastle upon Tyne, UK

**Kerry**, Raphael James, *Fellow*, Sheffield, UK

**Lipscomb**, Colin Francis, *Member*, Qualicum Beach, British Columbia, Canada

**Mahapatra**, Samarendra Nath, *Fellow*, Billingham, UK

**MacLean**, Rona, *Fellow*, Ashtead, UK

**Magapu**, Viswanathan, *Member*, Marchington, Staffordshire, UK

**Marks**, John, *Fellow*, Duxford, Cambridge, UK

**Martlew**, Robert Howard, *Fellow*, Wallasey, Merseyside, UK

**McGauley**, Gillian Anne, *Fellow*, London, UK

**McGregor**, Charles Malcolm, Foundation Affiliate, Doncaster, UK

**McPherson**, Angus Francis, *Member*, Findon, Worthing, UK

**McQuaid**, Arthur, *Fellow*, Cambridge, UK

**Middlefell**, Robert, *Fellow*, Raby Mere, Wirral, UK

**Milner**, George, *Fellow*, Worcester, UK

**Nazir**, Zoonia, *Member*, Birmingham, UK

**Noble**, Rochelle Fredelene, *Affiliate*, Salford, UK

**Nwulu**, Bernard Nchewa, *Fellow*, Rotherham, UK

**O'Brien**, James (Jim), Member, King's Lynn, Norfolk, UK

**O'Malley**, Patrick Pearse, *Fellow*, Booterstown, Co Dublin, Ireland

**O'Sullivan**, Mary Teresa Martin, *Member*, Baldrine, Isle of Man

**Pargiter**, Russell Ashby, *Fellow*, Hobart, Tasmania, Australia

**Prins**, Herschel Albert, *Mental Health Associate*, Houghton on the Hill, Leicester, UK

**Rae Grant**, Naomi Ingrid, *Fellow*, North York, Ontario, Canada

**Rae-Grant**, Quentin Alexander Frain, *Fellow*, Toronto, Ontario, Canada

**Read**, Priscilla Elise, *Fellow*, Layer Breton, Colchester, UK

**Rosen**, Bernard Keith, *Fellow*, London, UK

**Satkunanayagam**, Vaithianathan, *Fellow*, Epsom, UK

**Schnieden**, Vivienne, *Fellow*, Paddington, New South Wales, Australia

**Scott**, Jeremy Peter Dixon, *Member*, Lillte Petherick, Wadebridge, Cornwall, UK

**Silk**, Kenneth R., *International Associate*, Ann Arbor, Michigan, USA

**Silverman**, Maurice, *Fellow*, Whitefield, Manchester, UK

**Smith**, Alfred Leonard Gordon, *Fellow*, Lindley, Huddersfield, UK

**Speed**, Dorothy Elizabeth Maud, *Foundation Affiliate*, Rode, Frome, UK

**Thomas**, David John, *Member*, Dudley, West Midlands, UK

**Thomas**, Mira David, *Member*, Bathgate, West Lothian, UK

**Thompson**, Ruth, *Board of Trustees Committee Member*, London, UK

**Tychopoulos**, Gregory, *Member*, Attikis, Athens, Greece

**Walton**, John Nicholas, *Honorary Fellow*, Belford, UK

**Waters**, Thomas Cyril, *Member*, Newent, Gloucestershire, UK

**Wells**, Joan Catherine, *Member*

**Wilson**, Alan Hamilton, *Fellow*, Bessacarr, Doncaster, UK

**Younas**, Ayesha, *Affiliate*, Bolton, UK

**Zealley**, Andrew King, *Fellow*, Edinburgh, UK

